# A Pediatric Sepsis Protocol Reduced Mortality and Dysfunctions in a Brazilian Public Hospital

**DOI:** 10.3389/fped.2021.757721

**Published:** 2021-11-08

**Authors:** Daniela Nasu Monteiro Medeiros, Ana Carolina Cintra Nunes Mafra, Joseph Anthony Carcillo, Eduardo Juan Troster

**Affiliations:** ^1^Department of Pediatric Intensive Care Unit, Hospital Israelita Albert Einstein, São Paulo, Brazil; ^2^Center for Indicators and Information Systems, Hospital Israelita Albert Einstein, São Paulo, Brazil; ^3^University of Pittsburgh, Pittsburgh, PA, United States; ^4^Faculdade Israelita de Ciências da Saúde Albert Einstein, Hospital Israelita Albert Einstein, São Paulo, Brazil

**Keywords:** sepsis, protocol, emergence care, mortality, pediatrics, children, septic shock

## Abstract

**Introduction:** Few studies in the literature discuss the benefits of compliance with sepsis bundles in hospitals in low- and middle-income countries, where resources are limited and mortality is high.

**Methods:** This is a retrospective cohort study conducted at a public hospital in a low-income region in Brazil. We evaluated whether completion of a sepsis bundle is associated with reduced in-hospital mortality for sepsis, severe sepsis, and septic shock, as well as prevention of septic shock and organ dysfunction. Bundle compliance required the completion of three items: (1) obtaining blood count and culture, arterial or venous blood gases, and arterial or venous lactate levels; (2) antibiotic infusion within the first hour of diagnosis; and (3) infusion of 10–20 ml/kg saline solution within the first hour of diagnosis.

**Results:** A total of 548 children with sepsis, severe sepsis, or septic shock who were treated at the emergency room from February 2008 to August of 2016 were included in the study. Of those, 371 patients were included in the protocol group and had a lower median length of stay (3 days vs. 11 days; *p* < 0.001), fewer organ dysfunctions during hospitalization (0 vs. 2, *p* < 0.001), and a lower probability of developing septic shock. According to a propensity score analysis, mortality was lower during the post-implementation period [2.75 vs. 15.4% (RR 95%IC 0.13 (0.06, 0.27); *p* < 0.001)].

**Conclusions:** A simple and low-cost protocol was feasible and yielded good results at a general hospital in a low-income region in Brazil. Protocol use resulted in decreased mortality and progression of dysfunctions and was associated with a reduced probability of developing septic shock.

## Introduction

Sepsis prognosis depends greatly on time of diagnosis and treatment (1, 2). Sepsis has a 4.5–21% mortality rate, while septic shock mortality is 17–34% (3). Since 2002, the ACCM/PALS (American College of Critical Care Medicine—Pediatric Advanced Life Support) has published guidelines for the treatment of septic shock, but many studies show a low rate of adherence (1, 4, 5).

According to Tan et al., children with severe sepsis in developing countries are four times as likely to die than those in developed countries (6). Moreover, few studies discuss the benefits of compliance with sepsis bundles in hospitals in low- and middle-income countries, where resources are limited (7).

Here, we hypothesized that the complete application of sepsis bundles in a Brazilian hospital would result in reduced in-hospital mortality, a lower incidence of septic shock, and fewer cases of organ dysfunction during hospitalization.

In this article we aimed to evaluate whether completion of a sepsis bundle would result in: (1) reduced in-hospital mortality for sepsis, severe sepsis, and septic shock; and (2) prevention of septic shock and organ dysfunction.

## Methods

### Study Design

In this retrospective cohort study, we compared septic patients whose emergency room care adhered to a sepsis bundle protocol with patients whose treatment did not. We analyzed mortality, probability of septic shock, and organ dysfunction during hospitalization.

We used data collected from 2008 to August of 2016 in the database of the Quality Department and Medical and Statistics Archive Service of Hospital Dr. Moysés Deutsch—M'Boi Mirim (HMMD), in São Paulo, Brazil, a 300-bed general public hospital in a low-income area that serves a population of 650,000. HMMD was inaugurated in April of 2008.

### Definitions and Inclusion Criteria

Here, we defined sepsis, severe sepsis, septic shock, and organ dysfunction based on the criteria established by the International Pediatric Sepsis Consensus Conference of 2005 (IPSCC) (8), and treatment was determined based on the 2012 Surviving Sepsis Campaign (9).

During the pre-implementation period, we included patients that received one of the following ICD-10 (International Classifications of Diseases 10^th^ Edition) discharge codes (August 2008 to December 2014): A.41 (Septicemia), R.57 (Shock), or A.39 (Meningococcemia). We then excluded medical records that did not meet IPSCC criteria.

Then, during the post-implementation period (from February 2015 to August 2016), we included (1) patients included in the sepsis protocol; and (2) patients with ICD-10 discharge codes A.41, R.57, or A.39 who were not included in the protocol and met IPSCC criteria.

Bundle compliance to the sepsis protocol required the completion of three items: (1) obtaining blood count and culture, arterial or venous blood gases, and arterial or venous lactate levels; (2) antibiotic infusion within the first hour of diagnosis; and (3) infusion of 10–20 ml/kg saline solution within the first hour of diagnosis.

Blood cultures were collected after rigorous antisepsis with 70% alcohol, applied 3–4 times on the skin; 1–3 ml blood samples were placed in a Bac tec® flask (PEDS PLUS/F).

“Time Zero” was the time of hospital admission, and all times were recorded relative to time zero.

We define a chronic condition as any medical condition that persists for at least 12 months and that requires specialist follow-up (10). Our population included the following chronic conditions: chronic encephalopathy, convulsive syndrome, oxygen-dependent bronchopulmonary dysplasia, mechanical ventilation dependency, asthma, and bullous epidermolysis. At the time of data collection, we indicated the presence of chronic conditions as “Yes or No” and we did not collect any data related to diagnosis.

### Exclusion Criteria

We excluded patients who developed sepsis, severe sepsis, or septic shock in the Pediatric Intensive Care Unit, Neonatal Intensive Care Unit or Ward, and patients transferred from other services with sepsis, severe sepsis, or septic shock.

### The Protocol

The pediatric sepsis protocol was initiated at the HMMD pediatric emergency unit in February 2015. The protocol consisted of screening patients who presented with a history of fever or hypothermia at triage or first medical care with one of the following signs: tachycardia, tachypnea, hypotension, altered tissue perfusion, respiratory discomfort, oxygen saturation <92%, altered consciousness, or oliguria.

The medical chart was electronic, but the system could not alert if there were vital sign alterations. To facilitate the process, we placed a banner at each treatment location that showed the normal range of vital signs for different age groups. Then team could recognize the alterations of vital signs and star the protocol.

Whenever a protocol was opened, the triage nurse or physician at first medical care filled out a form. Author DNMM reviewed all forms with the help of a pediatric nurse. Next, they reviewed all patients with discharge codes ICD 10 A.41, ICD 10 R.57, and ICD 10 A.39 to identify patients with sepsis, severe sepsis, or septic shock who did not receive the protocol.

### Statistics

Patients from the pre-intervention period and those who were not treated according to protocol in the post-intervention period were grouped together in the “no protocol” group.

The variable times of fluid and antibiotic administration were categorized into: 1 h (0–60 min); 2 h (61–120 min); 3 h (121–180 min); 4 h (181–240 min); 5 h (241–300 min), and more than 5 h (more than 301 min).

To assess the association between death by septic shock and other variables in the patient's profile, we adjusted a Poisson regression model to consider the total length of hospital stay as the patient's follow-up time (11–13).

To obtain multiple models of the factors associated with septic shock and death, variables were selected following a stepwise process in both directions (14).

We used an adjusted Poisson regression model to measure growing sepsis protocol compliance.

We also estimated generalized mixed effects linear models to compare patients' clinical condition at the time of admission vs. hospital discharge (T0 and T1). The models considered the effect of the moment, protocol compliance and the interaction between the two factors. For septic shock, we used a logistic model with binomial distribution, and for the number of dysfunctions, we used a Poisson distribution with logarithmic connection. Confidence intervals were constructed using 1,000 simulations.

We used a propensity score matching analysis to assess the effect of adopting the protocol (15). Paired variables were patients' profile upon admission (i.e., age, gender, dysfunctions, and presence of chronic disease). Patients were excluded if their charts contained missing data (16). Analyses were conducted using the R 3.4.1 program with the aid of the PF, sandwich and *Matching* packages, and global significance was set at 5% (17, 18).

### Ethical Considerations

The project was approved by the Ethics and Research Committee (ERC) of the Municipal Health Department of São Paulo and Hospital Israelita Albert Einstein under protocols CAAE 55237316.3.0000.0086 and 55237316.3.3001.0071. The ERC of the Municipal Health Department of São Paulo usually asks researchers to obtain informed consent in retrospective studies. Because the study covered a long period of time, the ERC dispensed consent for parents who could not be reached and for those who did not attend the meeting.

### Patient and Public Involvement

At the beginning of the study, patients' parents received an invitation to participate in a meeting in which author DNMM explained what sepsis is, how to recognize clinical signs in children, and the study's significance. Afterwards, DNMM asked parents' consent to authorize chart analysis. During protocol implementation, DNMM also trained health professionals of the HMMD Health Network to disseminate knowledge about sepsis and to work toward earlier diagnosis and treatment.

## Results

**Figure 1:**
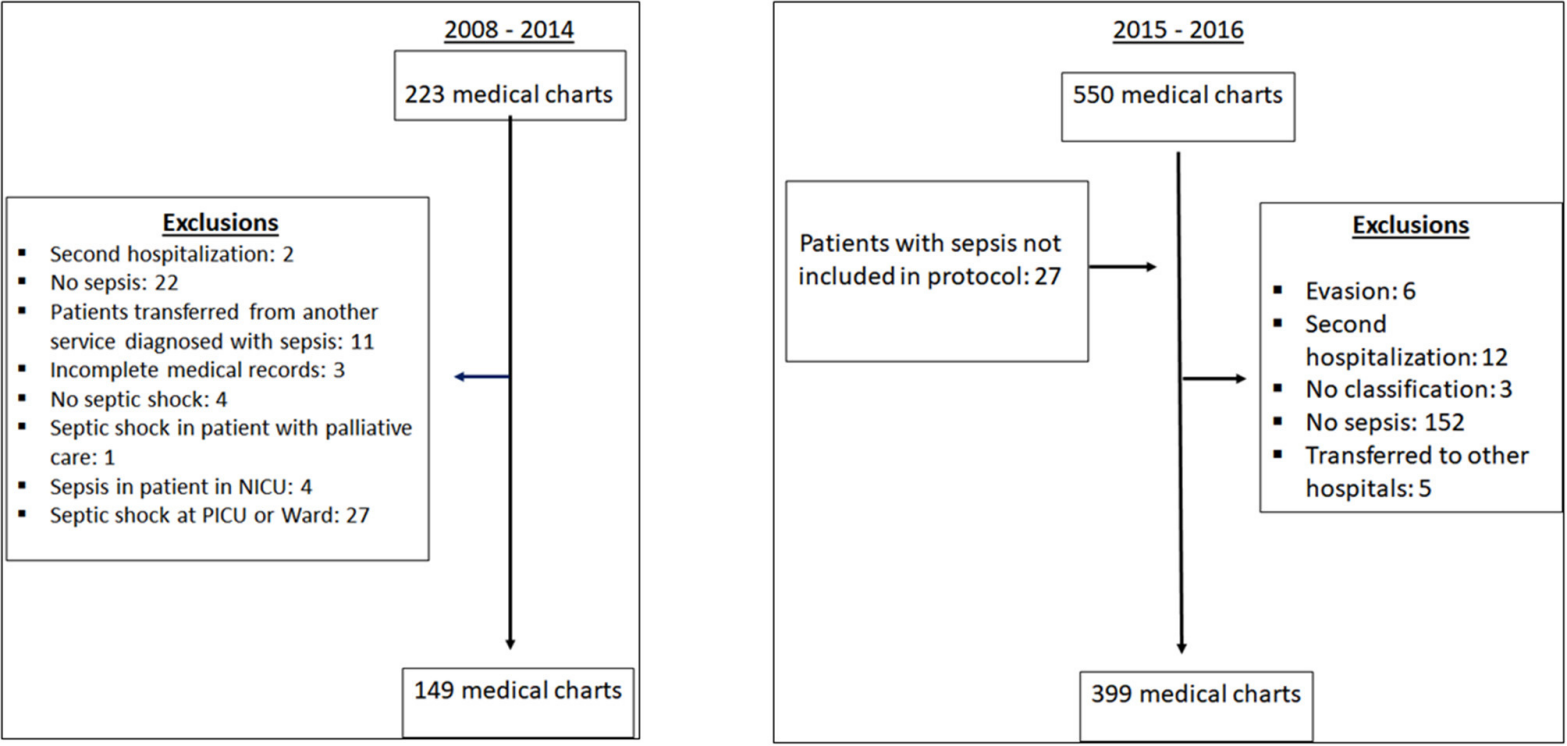
Flow chart.

Figure 1 shows the data collection process. During the post-implementation period, 27% of patients who screened positive (leading to notification) were subsequently deemed not to have sepsis. Those patients were excluded because vital signs were not altered according to protocol vital sign parameters, or because they were not sepsis patients. None of these patients had any complications associated with sepsis treatment and were discharged. This was common during the first semester of implementation.

Table 1 lists patient characteristics according to protocol compliance.

**Table 1:** Characteristics of patients included and not included in the protocol.

**Group**	**No protocol** **(177)**	**Protocol** **(***n*** = 371)**	***P*** **-Value**
Age, months [median, IIQ]	8.75 [2, 40.79]	19 [7, 50.5]	<0.001^*^
Gender, male (%)	101 (57.4)	195 (52.7)	0.35
Sepsis severity (%)			<0.001^*^
Sepsis	67 (37.9)	274 (73.9)	
Severe sepsis	29 (16.4)	91 (24.5)	
Septic shock	81 (45.8)	6 (1.6)	
Diagnosis (%)			
Pneumonia	73 (41.5)	121(32.6)	0.054
Bronchiolitis	33 (18.8)	57 (15.4)	0.382
Meningitis	15 (8.5)	11 (3)	0.008
Diarrhea	11 (6.2)	38 (10.2)	0.172
Asthma exacerbation	1 (0.6)	19 (5.1)	0.016
Chronic disease	21 (11.9)	24 (6.5)	0.047
Fever without localizing signs	1 (6.6)	35 (9.4)	<0.001^*^
Infectious agents (%)	42 (23.7)	46 (12.4)	0.001^*^
Respiratory syncytial virus	9 (5.1)	9 (5.1)	>0.999
Influenza virus	4 (2.3)	3 (0.8)	0.313
Adenovirus	3 (1.7)	0 (0)	0.058
*Streptococcus* sp.	6 (3.4)	4 (1.1)	0.34
*Staphylococcus* sp.	3 (1.7)	13 (3.6)	0.365
*E. coli*	10 (5.6)	5 (1.3)	0.009
Treatment			
Time to fluid administration (h) [median, IIR]	2 [1, 5]	3 [2, 6]	<0.001^*^
Time to antibiotic administration (h) [median, IIQ]	4 [2, 6]	3 [2, 6]	0.261
Time to vasoactive drug administration (h) [median, IIQ]	6 [3, 6]	6 [6, 6]	0.225
Outcomes			
Length of stay (days) [median, IIQ]	11 [4, 19.2]	3 [1, 6]	<0.001^*^
Admission to PICU (%)	112 (63.3)	38 (10.3)	<0.001^*^
Number of dysfunctions during hospitalization	2 [0, 3]	0 [0, 0]	<0.001^*^
Deaths (%)	27 (15.3)	3 (1.1)	<0.001^*^

*Categorical variables described by absolute frequency and percentage. IIQ, interquartile range. P-values of Mann-Whitney or Fisher tests. Considering 24 tests performed, the level of significance should be less than 0.002. N = 548*.

* *Significant values*.

### Probability of Developing Septic Shock and Organ Dysfunction During Hospitalization

**Figure 2:**
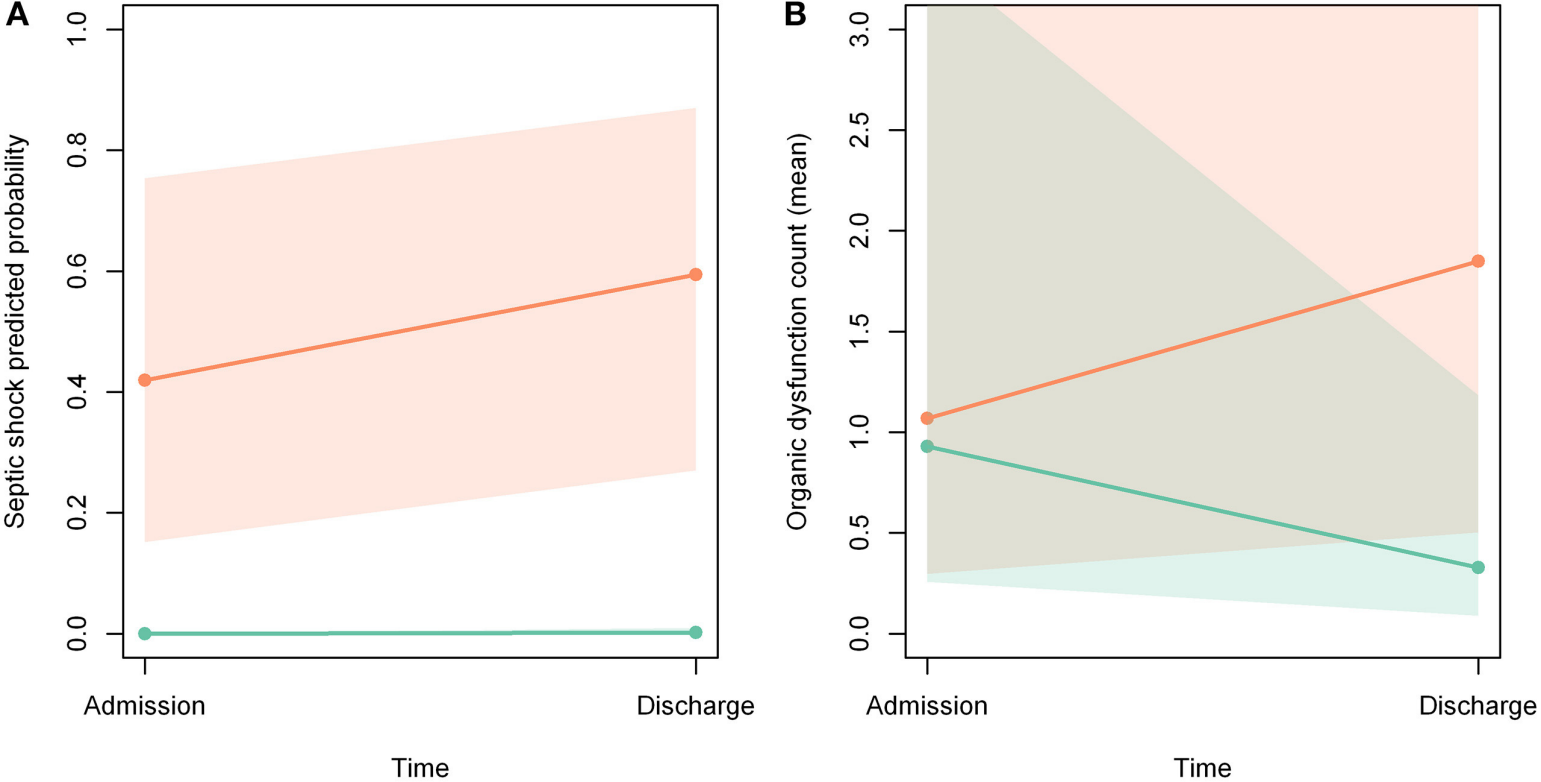
**(A,B)** Probability of developing septic shock and organ dysfunction during hospitalization. Orange line: patients not included in the protocol; green line: patients included in the protocol; orange area: confidence interval of patients not included; green area: confidence interval of patients included; gray zone: intersection of confidence intervals.

Figures 2A,B show that the probability of septic shock and number of organ dysfunctions increased during hospitalization among patients not included in the protocol. In Figure 2A there was no intersection of confidence intervals, so the difference reached statistical significance. In Figure 2B there was an intersection of the confidence intervals, so the difference did not reach statistical significance.

### Protocol Compliance

**Figure 3:**
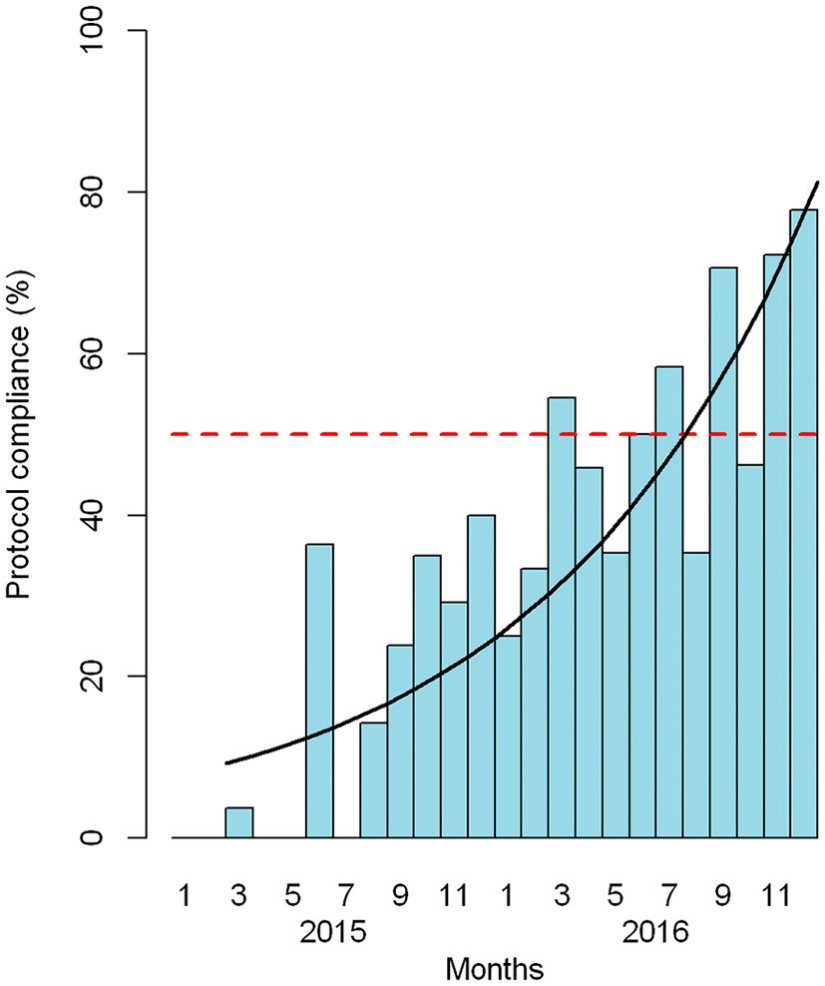
Adherence to the sepsis package among eligible patients by month. Red dashed line: 50% adherence. Black line: estimated growth of adherence.

Figure 3 shows the percentage of patients who complied with the protocol in relation to the total number of eligible patients. There was an overall growth rate of 1.1 (95% CI: 1.07, 1.14) per month after February 2015, or an increase of 10% each month relative to the previous month.

### Mortality

Table 2 lists the factors associated with sepsis-related deaths controlled only by length of hospital stay. It should be noted that both the 2015–2016 period and inclusion in the protocol were protective factors against mortality (*p*-values <0.001). All organ dysfunctions were significant risk factors for death, as well as the number of organ dysfunctions upon hospitalization and the number of new dysfunctions (all *p*-values <0.001). The presence of chronic disease and admission for septic shock were also risk factors for mortality (*p*-values < 0.001).

**Table 2:** Factors associated with mortality.

**Measures**	**Discharge** **(***n*** = 517)**	**Death** **(***n*** = 31)**	RR^*^	***P*** **-value**
Period			0.1	<0.001^*^
2008–2014	126 (24.4)	23 (74.2)	(0.04, 0.25)	
2015–2016	391 (75.6)	8 (25.8)		
Protocol	367 (71)	4 (12.9)	0.05 (0.02, 0.25)	<0.001^*^
Gender (*n* = 547)			0.78 (0.4, 0.15)	0.484
Male	281 (54.6)	16 (48.4)		
Age (months)			1 (0.99, 1.01)	0.492
Median [IIQ]	14 [5, 46]	16,1 [2, 48]		
Evolution				
Dysfunction during hospitalization	181 (35)	31 (88.6)		
Respiratory dysfunction	129 (25)	26 (83.9)	17.14 (6.51, 45.15)	<0.001^*^
Renal dysfunction	17 (3.3)	8 (26.7)	8.76 (3.8, 20.21)	<0.001^*^
Cardiovascular dysfunction	94 (18.2)	30 (96.8)	127.98 (17.42, 940.36)	<0.001^*^
Hematologic dysfunction	19 (3.7)	13 (41.9)	12.44 (6.47, 23.95)	<0.001^*^
Hepatic dysfunction (*n* = 545)	10 (1.9)	8 (26.7)	10.9 (5.23, 22.72)	<0.001^*^
Neurologic dysfunction	72 (13.9)	25 (80.6)	28.21 (12.03, 66.13)	<0.001^*^
Number of dysfunctions during hospitalization			2.32 (2.02, 2.66)	<0.001^*^
Median [IIQ]	0 [0, 1]	4 [3, 5]		
Number of new dysfunctions			2.08 (1.86, 2.33)	<0.001^*^
Median [IIQ]	0 [1, 0]	2 [2, 2.5]		
LOS (days) (*n* = 548)			1 (0.98, 1.03)	0.848
Median [IIQ]	4 [1, 11]	2 [1, 7.5]		
Chronic disease	37 (7.2)	8 (25.8)	3.89 (1.81, 8.35)	<0.001^*^
Septic shock	59 (11.4)	28 (90.3)	72.34 (22.57, 231.83)	<0.001^*^

*RR, relative risk of death estimated by Poisson models and controlled by hospital stay; IIQ, interquartile range (1st, 3rd quartiles). Considering 16 tests performed, the level of significance should be 0.003. LOS, length of stay*.

* *Statistical significance*.

During the post implementation period, 10 patients had septic shock. Four of these patients were not included in the protocol and all of them died. The remaining six patients were included in the protocol and three of them died. Among the six patients included in the protocol, two had five organ failures and one patient had four organ failures within the first 6 h of treatment.

Between the pre- and post-implementation periods, overall mortality decreased from 15.4 to 2.75% [RR 95%IC 0.13 (0.06, 0.27); *p* < 0.001].

Mortality was also assessed using the propensity score, matching age, gender, presence of chronic disease and number of dysfunctions between the pre- and post-implementation periods. The *p*-value was set at <0.001. The number needed to treat (NNT) with the protocol to prevent septic shock was 12 and the NNT with the protocol to prevent death was 8.

## Discussion

Protocol usage increased the number of sepsis diagnoses. Patients included in the pediatric sepsis protocol experienced reduced mortality and fewer new organ dysfunctions, which was mostly associated with a lower probability of developing septic shock. In septic patients with organ failures at admission, we did not observe reduced mortality.

Our study contributes to information regarding protocol usage in low-and middle-income countries. The implementation of a simple low-cost protocol was feasible and helped in the diagnosis of sepsis cases. Further, adherence increased over time.

According to Kortz et al., there are many challenges to data collection in resource-limited settings, such as a lack of research infrastructure, incomplete documentation, severe personnel shortages, inadequate funding, and technical difficulties performing follow-up (19). Even with these limitations, a considerable number of charts were revised with ICD-codes and IPSSC criteria.

One of our aims was to elaborate a low-cost protocol. We chose lactate as a bundle item because blood levels are a good marker of tissue hypoperfusion, septic shock adverse outcomes, and treatment improvement (20–22).

In the no-protocol group, there were fewer cases of sepsis and severe sepsis, which may be partly explained by the use of the ICD for screening. Balamuth et al. showed that different ICD screening methodologies differently influence prevalence and mortality data (23). Other authors also discuss that screening using ICD could under diagnosed sepsis (24–26). Pediatric and adult studies also reported an increase in sepsis and severe sepsis cases after protocol implementation (27, 28).

Because the Emergency Department initially applied the sepsis protocol to critically ill patients, many patients without sepsis and with signs of dysfunction due to other diseases (e.g., status epilepticus, intoxications, diabetic ketoacidosis) were included at the beginning of protocol implementation. No patient experienced adverse events due to protocol treatment. The inclusion of no sepsis patients was also observed by Kortz et al. after implementing the sepsis protocol in Bangladesh. Those authors reported that provider awareness increased and that there may have been a post-protocol diagnosis bias, with more children diagnosed with sepsis not meeting sepsis criteria. This may have also happened in our study (7).

The protocol group had a shorter LOS and fewer cases of new organ dysfunction during hospitalization, suggesting that patients were diagnosed at disease onset, which helped avoid further complications. Similar findings were reported by four studies conducted in the US: Arikan et al. observed lower acute kidney injury among patients included in their protocol (25, 29); in Balamuth et al., patients treated with protocolized emergency department care were more likely to be free of organ dysfunction on hospital day 2, at a tertiary care children's hospital (23); finally, Cruz et al. and Paul et al. also observed a reduction in LOS after protocol implementation (27, 30). In our study, protocol and no-protocol groups differed in terms of severity. To avoid overestimating the protocol effect, we applied the propensity score to analyze mortality, which was lower in the protocol group (*p* < 0.05). Following the New York sepsis care mandate, Evans et al. observed that mortality decreased among patients who received all three elements of a bundle within an hour, which included blood culture, antibiotics, and a 20 ml/kg isotonic fluid bolus (28, 31). Rodrigues-Santos et al. also observed a four-fold reduction in mortality after protocol implementation in a Children's referral Hospital in Rio de Janeiro, Brazil (29). In contrast, in a study conducted in a poor area of Bangladesh, Kortz et al. did not observe increased compliance or a reduction in median times to fluid and antibiotic administration, LOS, or mortality (7). Those authors reported an increase in cases of fluid overload and heart failure in the post protocol group, which we did not observe in our protocol group.

It should be noted that differences in mortality between the pre- and post-implementation periods may be due in part to improvements in immunization, socioeconomic status, nutrition, sanitary conditions, and quality of health care over the years, and that we did not analyze these variables in the current study.

In our study, we did not observe reduced mortality among septic patients with organ failures at admission. Kortz et al. described clinical presentations of children with sepsis in Tanzania and observed that 80% of children who died within 48-h received a fluid bolus and 95% received antibiotics either pre-arrival or in the Emergency Department. They concluded that in this subgroup, closely following guidelines failed to alter disease progression (19). De Souza et al. conducted a *post-hoc* analysis of the Latin American Pediatric Sepsis Study (LAPSES) data and observed that the prevalence of septic shock within the first 24 h of pediatric ICU admission and sepsis-related mortality was significantly higher in public hospitals relative to private hospitals. The authors attributed this difference to greater disease severity and a larger proportion of patients with prior comorbidities and septic shock on admission in public hospitals (32). In our study, other factors also significantly contributed to death, such as chronic diseases, septic shock at hospital admission, and new organ dysfunction during hospitalization.

The fact that septic shock patients had several organ failures at admission may reflect a late arrival at the hospital. In Launay et al., causes of death in children with severe bacterial infections were associated with parental delay in seeking medical care (33). Kang et al. surveyed 101 hospitals in 41 countries and observed that a lack of knowledge among parents concerning the early recognition of sepsis is one of the biggest barriers to treatment (34). A previous study found that the Brazilian public in general has little knowledge about sepsis (35). Furthermore, the population around the hospital is socially vulnerable and “sepsis zero hour” may have happened at home. For this reason, we defined time zero as the moment the patient was admitted to the hospital reception and not when sepsis was suspected. This metric was also adopted by Long et al. in a study conducted in Melbourne, Australia (36). We did not collect data about the days of disease at time zero, which would clarify the cause of severity on admission. It is a limitation of our study and could be an object of future studies. Jaramillo-Bustamante et al. (Colombia), de Souza et al. (Brazil), and Gavidia et al. (El Salvador) observed that low income, illiteracy, and low maternal education level was associated with sepsis mortality (37, 38).

According to Melendez, the definition of time zero is central for comparing between studies but there is no standardization so far (39). Since we considered patient admission as time zero and collected time at triage before the first medical care, our median volume and antibiotic times are longer when compared with studies that considered time zero as the time of recording exams, clinical deterioration, or the time of triage (27, 40, 41). One limitation of adopting this definition is losing patients who developed sepsis outside the Emergency Department.

The median time to fluid administration was longer in the protocol group. Crystalloids are available for administration in the ED as soon as the medical order is given and this may not have been properly documented. This difficulty was also faced by Kortz et al. (7). Conn et al. conducted an observational study that analyzed critical incident reports relating to intravenous fluid prescribing errors in children aged 0–16 in secondary care in the United Kingdom. The incorrect completion of the fluid prescription chart was the third most common cause of errors (42). Second, the more severe patients in the no-protocol group may have received more prompt interventions. Cruz et al. also observed that children in the shock protocol received interventions more rapidly and with less variations than the patients with sepsis (27).

We observed good results even with limited resources, as our hospital does not have an alert system for vital sign screening, or a team dedicated to sepsis treatment. Thus, protocol was initiated based on clinical signs. This simple and low-cost protocol could be applied in low-income countries with good results.

Our study has limitations. First, it was conducted in a single center; thus, more data are needed from other centers to corroborate our findings. Second, no severity score was applied upon admission to the Pediatric ICU. Third, we did not record the localization of discharge from the emergency room: home, ward, or PICU. Fourth, we did not include ICDs of lower incidence diseases such as toxic shock.

Future studies should focus on the following issues: (1) protocols that restrict fluid administration; (2) protocols that do not consider laboratory exams as part of the protocol bundle; (3) barriers to adopting and implementing sepsis protocols, and (4) the development of efficient sepsis protocols designed especially for limited resource settings and specific populations.

## Conclusions

In our study, a simple low-cost protocol was feasible and yielded good results at a general hospital in a low-income region in Brazil. The protocol helped diagnose sepsis at earlier stages, which helped avoid further complications. Protocol use resulted in decreased mortality and progression of dysfunctions, which was associated with a reduced probability of developing septic shock.

## Data Availability Statement

The datasets presented in this study can be found in online repositories. The names of the repository/repositories and accession number(s) can be found below: doi: 10.17632/hj2j3drw3v.3 Description: Completion of sepsis bundle is associated with reduction in in-hospital mortality, in probability of septic shock development, and in organ dysfunctions on hospitalization.

## Ethics Statement

The studies involving human participants were reviewed and approved by the Ethics and Research Committee of the Municipal Health Department of São Paulo and Hospital Israelita Albert Einstein under protocols CAAE 55237316.3.0000.0086 and 55237316.3.3001.0071. Written informed consent to participate in this study was provided by the participants' legal guardian/next of kin.

## Author Contributions

DM, JC, and ET designed the study. DM collected the data and wrote the manuscript. AM conducted the statistical analysis and helped with methods. ET, AM, and JC reviewed and corrected the paper. All authors contributed to the article and approved the submitted version.

## Conflict of Interest

The authors declare that the research was conducted in the absence of any commercial or financial relationships that could be construed as a potential conflict of interest.

## Publisher's Note

All claims expressed in this article are solely those of the authors and do not necessarily represent those of their affiliated organizations, or those of the publisher, the editors and the reviewers. Any product that may be evaluated in this article, or claim that may be made by its manufacturer, is not guaranteed or endorsed by the publisher.
